# Integrating Cellular Metabolism into a Multiscale Whole-Body Model

**DOI:** 10.1371/journal.pcbi.1002750

**Published:** 2012-10-25

**Authors:** Markus Krauss, Stephan Schaller, Steffen Borchers, Rolf Findeisen, Jörg Lippert, Lars Kuepfer

**Affiliations:** 1 Bayer Technology Services GmbH, Computational Systems Biology, Leverkusen, Germany; 2 Aachen Institute for Advanced Study in Computational Engineering Sciences, RWTH Aachen, Aachen, Germany; 3 Laboratory for Systems Theory and Automatic Control, Institute for Automation Engineering, Otto-von-Guericke University, Magdeburg, Germany; 4 Institute of Applied Microbiology, RWTH Aachen, Aachen, Germany; University of Virginia, United States of America

## Abstract

Cellular metabolism continuously processes an enormous range of external compounds into endogenous metabolites and is as such a key element in human physiology. The multifaceted physiological role of the metabolic network fulfilling the catalytic conversions can only be fully understood from a whole-body perspective where the causal interplay of the metabolic states of individual cells, the surrounding tissue and the whole organism are simultaneously considered. We here present an approach relying on dynamic flux balance analysis that allows the integration of metabolic networks at the cellular scale into standardized physiologically-based pharmacokinetic models at the whole-body level. To evaluate our approach we integrated a genome-scale network reconstruction of a human hepatocyte into the liver tissue of a physiologically-based pharmacokinetic model of a human adult. The resulting multiscale model was used to investigate hyperuricemia therapy, ammonia detoxification and paracetamol-induced toxication at a systems level. The specific models simultaneously integrate multiple layers of biological organization and offer mechanistic insights into pathology and medication. The approach presented may in future support a mechanistic understanding in diagnostics and drug development.

## Author Summary

Cellular metabolism is a key element in human physiology. Ideally the metabolic network needs to be considered within the context of the surrounding tissue and organism since the various levels of biological organization are mutually influencing each other. To mechanistically describe the interplay between intracellular space and extracellular environment, we here integrate the genome-scale metabolic network model HepatoNet1 at the cellular scale into physiologically-based pharmacokinetic models at the whole-body level. The resulting multiscale model allows the quantitative description of metabolic behavior in the context of time-resolved metabolite concentration profiles in the body and the surrounding liver tissue. The model has been applied to three case studies covering fundamental aspects of medicine and pharmacology: drug administration, biomarker identification and drug-induced toxication. Most notably, our multiscale approach fosters an improved quantitative understanding of drug action and the impact of metabolic disorders at an organism level, based on a genome-scale representation of cellular metabolism. Computational models such as the one presented include various aspects of human physiology and may therefore significantly support rational approaches in medical diagnostics and pharmaceutical drug development in the future.

## Introduction

Human metabolism is an integral component of whole-body physiology and its dysfunction plays a key role in many systemic diseases. Frequent symptoms of metabolic diseases are changes in exometabolism [1], [2] which usually follow upstream alterations in intracellular flux distributions [3]. In order to associate diagnostic observations at the organism level accompanying specific diseases with structural impairment at the cellular level, a mechanistic understanding of genotype-phenotype correlations is essential [3], [4]. Adequate analytical methods for a systemic consideration of the underlying processes are still missing. However, such multiscale approaches are necessary to understand the highly complex and intertwined structure of biological networks and the interplay with the surrounding organism [3], [5], [6].

In recent years, modeling approaches have been developed describing biological processes at different levels of physiological organization based on multiple, divergent mathematical formalisms [5], [7], [8], [9]. At the whole-body level, physiologically-based pharmacokinetic (PBPK) modeling quantitatively describes the absorption, distribution, metabolization and excretion (ADME) of endogenous and exogenous compounds within mammalian organisms [10], [11], [12], [13]. In contrast to classical pharmacokinetic (PK)/pharmacodynamic (PD) modeling [14], PBPK models aim for a mechanistic representation of ADME-related processes. Structurally, PBPK models consist of compartmental representations of all relevant tissues and the vascular system. Most notably, PBPK models are based on large amounts of prior anatomical and physiological information as well as generic distribution models, such that most model parameters can be either obtained from database collections integrated in the modeling software or they can be deduced from the physicochemistry of the compound [15], [16], [17], [18], [19]. Hence, even though PBPK models contain more than hundred ordinary differential equations and several hundred variables, the number of independent parameters which need to be adjusted during model development is small (usually less than 10, see also Materials and Methods). ADME-related processes can automatically be quantified based on compound-deduced parameters allowing a detailed representation of mass transfer across various tissue compartments. PBPK models have previously been used for mechanistic analyses of drug pharmacokinetics [20], pharmacogenomics [21], species extrapolation [22] or analysis of rare adverse events [23].

For analyses at the cellular level, metabolic network reconstructions are an important tool of bottom-up systems biology. Cellular metabolism gathers a multitude of upstream regulatory events onto the various layers of cellular organization such as the transcriptome and metabolome representing an important angle point in the physiology of an organism. Metabolic networks are typically described by stoichiometric matrices and intracellular flux distributions are inherent variables in such models. First stoichiometric models on human metabolism at genome-scale encompassed generic collections of metabolic biochemistry in human cells [24], [25]. Recent models explicitly account for network structure in specific tissues thereby enabling, for the first time, the consideration of metabolic models within a specific context of human physiology [4], [26].

While metabolic network models are applicable to the investigation of *in vitro* experiments with more or less well-defined media conditions, they do not suffice for considerations of *in vivo* metabolism, where the cell is embedded in the ever-changing environment of the surrounding tissue and organism. Therefore, human metabolism can only be fully understood by an integrative analysis which simultaneously considers the whole-body context. This allows in particular the quantification of cellular boundary conditions and the interference with intracellular states and processes. Several approaches for combining models covering different levels of biological organization have been described before [8], [27], [28]. With regard to metabolic networks, dynamic flux balance analysis (dFBA) has been used to couple stoichiometric models of metabolism with dynamic models of microbial batch cultures and integrated omics networks [29], [30], [31].

**Figure 1:**
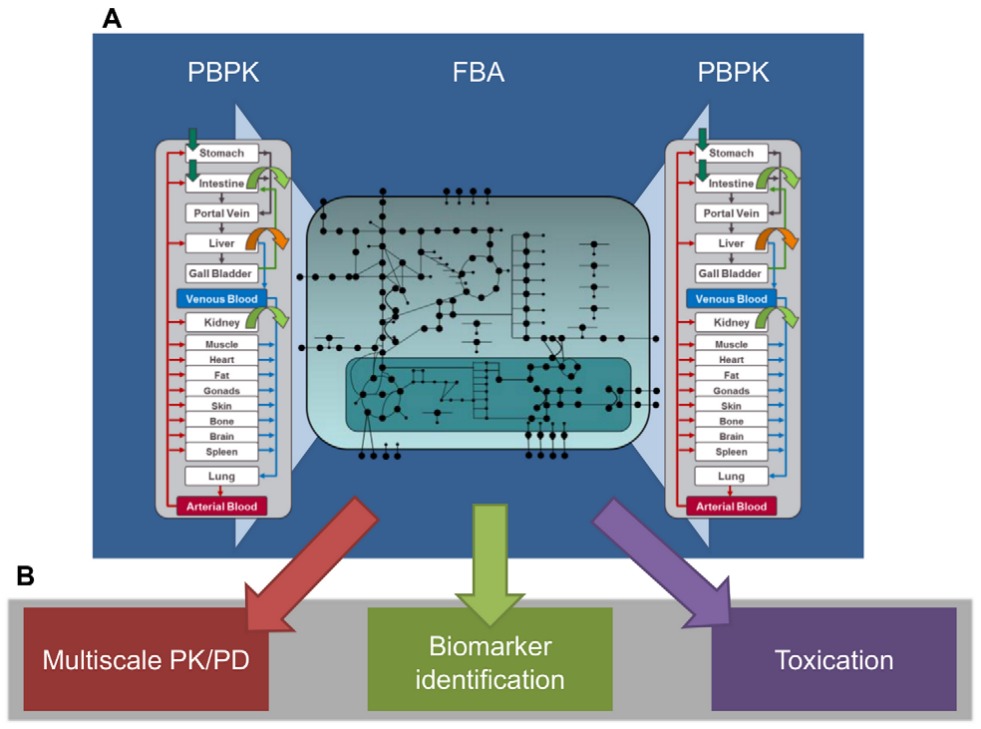
A bow-tie scheme illustrating the consideration of metabolic networks within a whole-body context. (**A**) Schematic representation of the multiscale approach. PBPK models are used to quantitatively describe the tissue specific availability of exogenous and endogenous compounds at the organism level. The PBPK models are coupled with stoichiometric networks by means of exchange rates calculated with dFBA. Both consumption and formation of metabolites as well as regulatory effects can be simulated. (**B**) Possible fields of application, illustrating the broad applicability of the approach, are (i) multiscale PK/PD modeling, (ii) quantitative identification of disease specific or individualized biomarkers and (iii) analyses of drug-induced toxication.

We here apply dFBA to describe human metabolic networks within the context of whole-body PBPK models (Figure S1 in Text S1). The approach allows the representation of human metabolism under simultaneous consideration of quantitative availability of substances at the organism level (Figure 1 A). We exemplarily use HepatoNet1 [26], a genome-scale model of human hepatic metabolism to analyze specific responses of the network in the face of time-dependent concentration profiles in liver tissue. Following this approach we investigate three application examples (Figure 1 B). First, we use a multiscale PK/PD model to analyze the distribution and therapeutic effect of allopurinol in the treatment of hyperuricemia. In a second example, we consider the effect of impaired ammonia metabolism on blood plasma levels to demonstrate the methods' capability to identify biomarkers specific for pathologic changes in the metabolic state [25], [32], [33]. Finally, we apply our approach to the analysis of paracetamol-induced toxication on liver function.

## Results

### Model coupling

PBPK models describe the processes underlying the distribution of a compound within the body based on prior physiological information and generic distribution models. Organs in PBPK models are usually subdivided in further compartments such as the vascular, interstitial and intracellular space [12], [34]. The basic differential equations within these compartments describe uptake, secretion, formation and consumption of a particular compound, representing overall mass balance equations [10]. In contrast, stoichiometric models describe mechanisms within the cell at a much finer spatial scale, providing a more detailed insight in intracellular processes with a particular focus on cellular biochemistry. Thus, the intuitive point of contact between both model formalisms is the intracellular space, where PBPK models quantitatively describe time-concentration profiles of endogenous or exogenous compounds, which in turn represent substrates or products of metabolic networks at the molecular level.

In order to relate the distribution of endogenous and exogenous compounds at the organism level to metabolic network structures and thus to a specific enzymatic process at the cellular level, the stoichiometric network was embedded in the dynamic whole-body model by step-wise model discretization. To this end, functional adaptation of metabolism, ultimately quantified by intracellular flux distributions and extracellular exchange rates, can be assumed to be fast in relation to the surrounding distribution processes at the whole body scale. Consequently, flux distributions are kept constant over each time interval [29], [30], [31]. In our case, the chosen time interval was 1 step/min. Hence, for a specific distribution of extracellular concentrations at a given point in time, intracellular steady state (i.e. equilibrium) can be assumed, and flux balance analysis (FBA) can be applied for the estimation of flux distributions [31].

Following the rational of network validation as used in HepatoNet1 [26] we here applied case-specific objectives such as maximization of ammonia production or maximization of uric acid production to quantify extracellular exchange rates with regard to a specific set of boundary conditions. Notably, intracellular flux distributions of biological relevance can hardly be identified using these functional objectives since they rather evaluate the macroscopic behavior of the cell. In contrast, the underlying flux space is assessed qualitatively. In our approach, a compound in the PBPK model can act either as a regulatory modifier or as a substrate of an enzymatic reaction in the metabolic network. We therefore considered two distinct ways of coupling PBPK models and stoichiometric network models: (1) indirect coupling, where concentrations of a compound in the PBPK model impose a regulatory effect on enzyme activity which is quantified at the cellular level (‘feed-forward’), thereby restricting fluxes through this specific reaction and (2) direct coupling, where perturbed metabolic processes (for instance inhibited enzymes) iteratively affect availability of a substance in the PBPK model by directly interfering the corresponding mass balance (‘feed-back’). In both cases, the intracellular concentration of a compound constrains a metabolic state in the underlying network structure [35]. This also influences further downstream events, since the catabolic or anabolic products formed within the intracellular metabolic network are again distributed at the whole organism level. This centralized consideration of metabolism as a core component in human physiology can be seen as an hourglass or bow-tie scheme (Figure 1 A) [36], [37]. In particular, enzymatic blockage results in accumulation of the upstream substrate, depletion of the downstream product and potential activation of alternative pathways. Details for indirect and direct coupling will be explained in the following.

#### Indirect coupling

Indirect coupling is used for the simulation of regulatory modifications such as drug-induced inhibition of metabolic enzymes (Equation 1, 2). In this case the inhibition of the affected enzyme has no effect on the concentrations of the acting drug. Thereby it is possible to describe the relative change of enzymatic activity and thus the change in an intracellular flux in the metabolic network as a consequence of time-resolved intracellular drug concentrations in a specific kind of tissue. Inhibitory rate laws as used for Michaelis-Menten kinetics are used for constraining a specific intracellular flux:
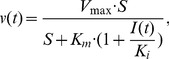
(1)

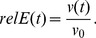
(2)Here, the concentration of the drug at a specific time point in the PBPK model corresponds to the inhibitor concentration *I(t)*. The relative enzyme activity (*relE(t)*) is the time-resolved ratio of the inhibited reaction rate *v(t)* and the uninfluenced reaction rate *v_0_* (*I = 0 µM*). Next, *relE(t)* is used to quantify the effect of enzyme inhibition in the metabolic network. The additional constraint for the FBA step imposed on the specific flux in the metabolic network is defined as:
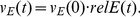
(3)In equation 3, *v_E_(t)* corresponds to the flux through the affected enzyme at each time step which is defined as the product of the unperturbed flux *v_E_(0)* which is calculated by FBA and the relative enzyme activity *relE(t)*. Therefore, the set of constraints changes over time such that the extracellular exchange rates identified with FBA also evolve dynamically. This allows on the one hand quantifying the effect of compound-induced extracellular perturbations on metabolic states. On the other hand, time-resolved exchange fluxes determined with FBA may interfere with the mass balance of the PBPK model such that direct coupling needs to be considered (see section below).

#### Direct coupling

**Figure 2:**
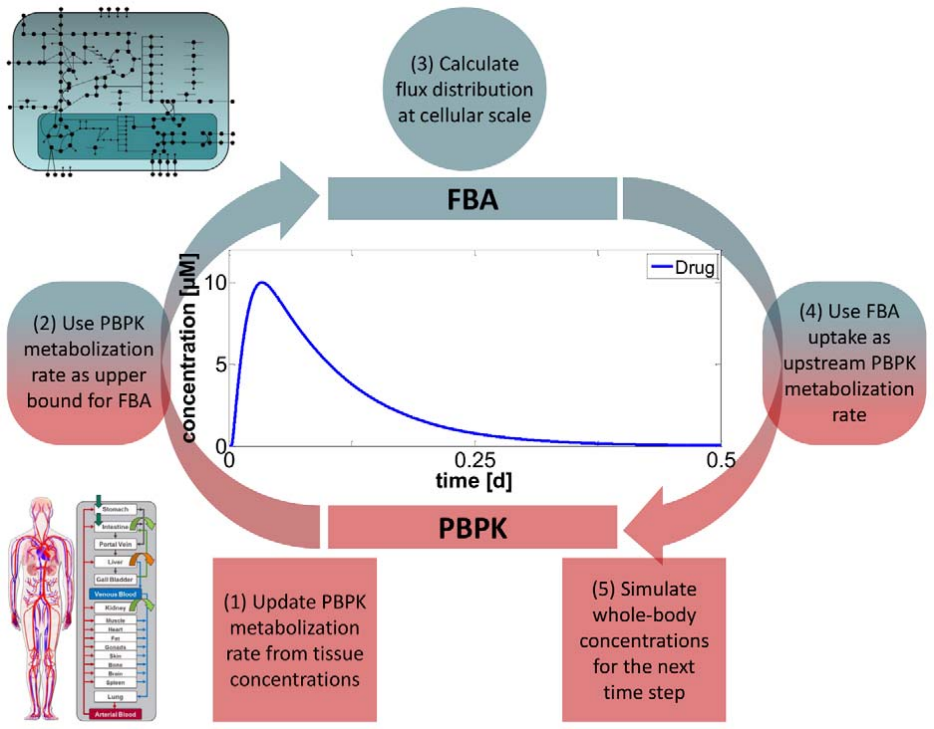
Schematic representation of the feed-back loop used for direct coupling. Flux distributions calculated by FBA are used to adjust clearance and production rates in the PBPK model. After simulating one time step in the PBPK model, new clearance rates constrain the next FBA step.

Metabolic clearance and formation processes are simulated by direct coupling since they directly interfere with the mass balance of the PBPK model. Direct coupling therefore describes the influence of active processes at the cellular level on the distribution of compounds at the whole-body scale. To this end, a feedback update loop is considered which consists of the following steps (Figure 2): (1) The clearance and production rates are first calculated by kinetic rate laws of the PBPK model. (2) The rates are subsequently used as upper bounds for FBA to (3) identify an intracellular flux distribution quantifying cellular exchange rates. (4) These exchange rates are then used as clearance and production rates in the PBPK model in order to integrate the next time step. (5) This results in new concentration levels at the end of the time-step, which are then again used to calculate the clearance and production rates for the next iteration (1).

While the exchange rates in the PBPK model and metabolic network are identical for the metabolic reference state, these rates will differ if internal and external network perturbations are imposed. Without any impairment of enzyme reactions in the metabolic network, the clearance or production rates calculated by FBA will reach the upper bound (step 2). In this case, the rates of the coupled model are identical to those of the PBPK model alone. In contrast, the rates calculated by FBA will decrease if for instance enzyme activities are impaired due to restricted functionality or structural network errors (Equation 3). Consequently the rates of the coupled model are decreased and this subsequently affects the concentrations at the whole-body scale (step 4).

### Allopurinol treatment

As a first example we analyzed drug action of allopurinol in the treatment of hyperuricemia with a multiscale PK/PD model. Purine metabolism provides a large number of drug targets [38], [39] with uric acid being the final downstream degradation product in the human body. Quantitatively modeling the effect of drugs affecting this crucial metabolic pathway therefore provides valuable insights for drug development.

In clinical practice, high plasma levels of uric acid (above 470 µM) are referred to as hyperuricemia which may result from inborn errors of purine metabolism and even more often from impaired renal excretion of uric acid, which is considered in the following. Hyperuricemia can lead to diseases like gout where uric acid is deposited into tissues, especially in the joints [40]. A currently used drug against hyperuricemia is the purine analog allopurinol [41], which reduces production of uric acid by inhibition of xanthine oxidase. This enzyme oxidizes hypoxanthine to xanthine and subsequently xanthine to uric acid [42]. Allopurinol itself is oxidized by xanthine oxidase to oxypurinol, which also inhibits xanthine oxidase as an active metabolite (Figure S2 in Text S1). Since metabolization of allopurinol to oxypurinol is very fast, but excretion of oxypurinol is very slow, oxypurinol plays a significant role in the inhibition of xanthine oxidase [41].

**Figure 3:**
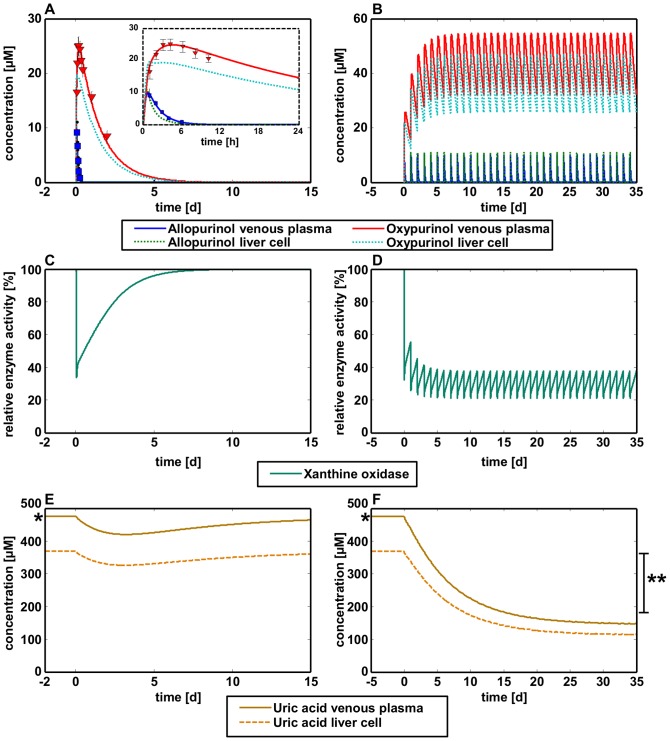
Reduction of uric acid production following multiple allopurinol administrations. (**A**) Simulated venous plasma and intrahepatic concentration profiles of allopurinol and oxypurinol are in agreement with experimental PK data [43]. (**B**) Prediction of venous plasma and intrahepatic concentration profiles of allopurinol and oxypurinol after multiple dosing based on the single application model. (**C**) Relative enzyme activity of xanthine oxidase (XO) following inhibition by a single dose of allopurinol. (**D**) Relative enzyme activity of XO following inhibition by multiple administration of allopurinol. (**E**) Simulated venous plasma and intrahepatic concentration profiles of uric acid following a single dose of allopurinol. (**F**) Simulated venous plasma and intrahepatic concentration profiles of uric acid following multiple dosing of allopurinol. Experimentally-measured venous plasma concentrations quantifying the hyperuricemic state (*) and the healthy uricemic state (**) [48] after treatment with allopurinol are indicated.

To estimate the inhibitory concentration of allopurinol and its active metabolite oxypurinol, a coupled PBPK model was developed (see Materials and Methods). Experimentally measured plasma concentrations following oral administration of 200 mg allopurinol [43] were considered for identification and fine-tuning of four physiological parameters describing the absorption and clearance of both compounds as well as four physicochemical parameters of allopurinol and oxypurinol (Section parameter identification in Text S1, Table S1 in Text S1). Notably, these parameters are distributed in the two independent PBPK models which are coupled by the clearance reaction. Exemplarily, a sensitivity analysis was performed to estimate the influence of each parameter to the model (Figure S3 in Text S1). The resulting coupled model simultaneously described the PK of allopurinol and oxypurinol with excellent accuracy (Figure 3 A). Having established a working model for single dosing of allopurinol, the PK of multiple administrations was predicted in the next step (Figure 3 B). When simulating concentration profiles of allopurinol and oxypurinol over a time range of 35 days it became obvious that allopurinol is not accumulating in the human body but is always completely degraded before the next application is given. In contrast, oxypurinol can no longer be completely removed from the body leading to mean oxypurinol concentrations of about 45 µM.

In the next step, a PBPK model for uric acid was created, which allowed describing changes in uric acid concentration as a mechanistic consequence of allopurinol inhibition in hepatic purine metabolism (Table S2 in Text S1). The plasma concentration of uric acid in healthy male individuals is around 302 µM (male: 302±60 µM, female: 234±52 µM), while patients with gout show much higher concentrations of approximately 480 µM [44]. This physiological information was used to identify three parameters for the steady state clearance and production rate of uric acid in a whole-body model of a healthy as well as a gouty male individual (Table S2 in Text S1). An identical uric acid production rate was assumed for both healthy and gouty individuals, since impaired renal excretion is assumed to be the physiological cause for hyperuricemia in the present case.

To finally couple the upstream distribution of allopurinol and oxypurinol at the whole-body scale with the subsequent inhibitory effect on xanthine oxidase in the hepatic metabolic network, indirect coupling was used to simulate the PD effect of a single as well as multiple allopurinol doses (Figure 3 C, D). Maximization of uric acid production was considered as objective function, and the resulting FBA problem was additionally constrained by drug-induced enzymatic inhibition as well as the uric acid formation rates estimated in the PBPK model. *IC50* values of allopurinol (13.4 µM [45]) and oxypurinol (15.6 µM [46]) were used to estimate the *Ki* constants of the enzyme inhibition by using the Cheng-Prusoff equation (Equations S1–S6 in Text S1) [47]. Taken together, the overall multiscale model comprised the two coupled whole-body PBPK models of allopurinol and oxypurinol, hepatic metabolism and the downstream whole-body PBPK model of uric acid.

After successful establishment of the multiscale model, the development of the uric acid level in gouty patients monitoring the therapeutic success of gout treatment after single and multiple dosing of allopurinol was simulated (Figure 3 E,F). Before allopurinol treatment, the mean gouty patient in hyperuricemic steady state had a venous plasma concentration of 476 µM. After a single application of allopurinol, uric acid concentrations began to decrease. Therapy interruption or patient non-compliance, however, led to a recovery of uric acid concentrations which are typical for the hyperuricemic state (Figure 3 E). Only when multiple dosings of allopurinol were routinely taken, a continuous decrease in uric acid concentrations could be achieved with venous blood concentrations of 146 µM. Most notably, the predicted uric acid concentration after multiple dosings was close to the range of uric acid observed *in vivo* (Figure 3 F) [48], [49] although the PBPK model was established based on a single dosing of allopurinol. This correct prediction of our model clearly emphasizes the predictive capabilities of the coupled model.

### Ammonia detoxification

As a second example we analyzed pathogenesis of urea cycle disorders at the cellular scale and the resulting effect on ammonia plasma concentration at the organism level. In particular we aimed for an evaluation of diagnostic markers for a specific health state or disease progression. Hepatic metabolization of amino acids and detoxification of ammonia play an important role in the human body. Up to 95% of ammonia metabolized in hepatocytes is degraded to urea, which is subsequently excreted, while about 5% are metabolized to glutamine and about 1% to alanine [26]. Impairment in ammonia metabolism leads to decreased ammonia elimination and thereby induces hyperammonemia [50]. A consequence of hyperammonemia is an increase of ammonia concentration in the brain - so-called hepatic encephalopathy - which can cause confusion, lethargy, disorientation and in severe cases coma and death [51], [52]. Liver dysfunction in ammonia metabolism can be caused by liver diseases or inborn errors of metabolism (IEMs), e.g. urea cycle disorders (UCDs), which may have lethal consequences without adequate treatment and diet [53]. Perturbations in ammonia detoxification capacity causes direct downstream changes in blood metabolite concentrations making ammonia detoxification a primary example for the identification of disease specific biomarkers.

In humans, ammonia is produced by the breakdown of amino acids in the liver or intense muscle exercise [54]. In addition to endogenous ammonia, exogenous ammonia also enters the body with nutritional intake. Altogether, about 17 g of ammonia are produced by the body every day. The excretion through the kidneys is about 13 g per day, while 4 g per day are metabolized by the liver. Furthermore, during impaired ammonia detoxification following UCDs, the rates of glutamine and alanine synthesis are increased 4–6 fold [53], [55].

**Figure 4:**
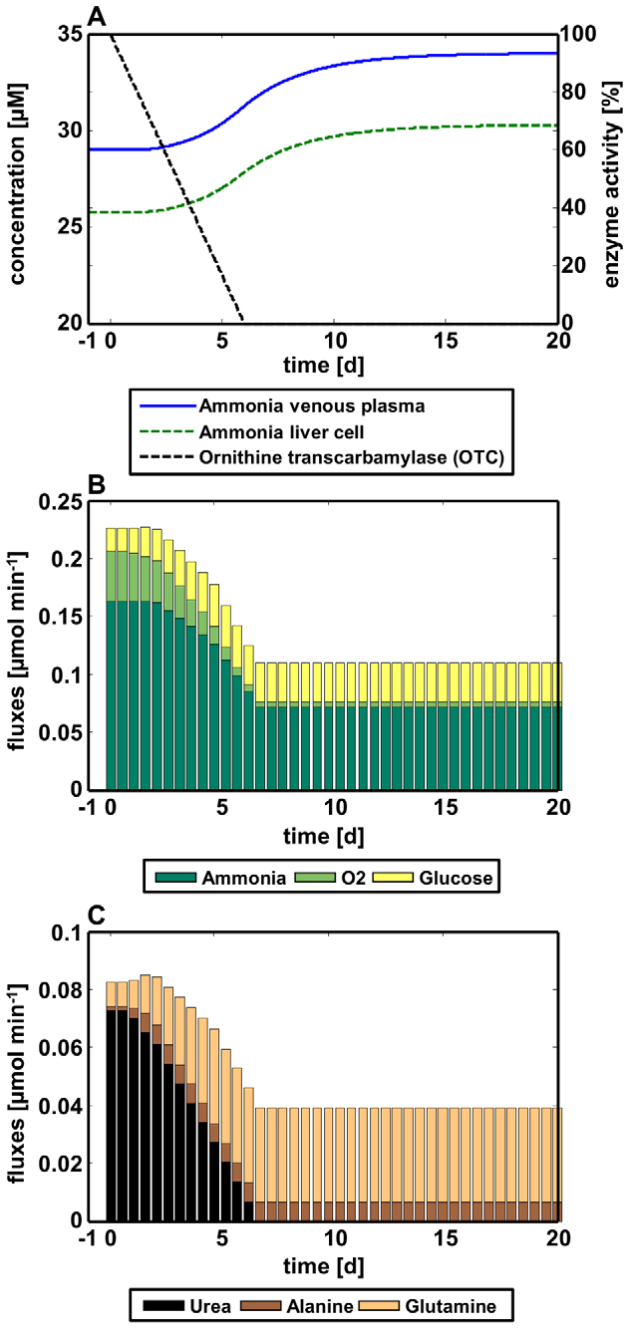
Pathogenesis of a urea cycle disorder in ammonia plasma concentrations and metabolic exchange rates. (**A**) Simulated venous plasma and intrahepatic concentration profiles of ammonia during development of a urea cycle disorder. The black dashed line represents the overall reduction in ornithine transcarbamylase activity. (**B, C**): Resulting exchange fluxes calculated with FBA during development of a urea cycle disorder. (B) Hepatic influx rates of the three substrates ammonia, oxygen and glucose. (C) Hepatic efflux rates of the three products urea, alanine and glutamine.

As a first step to investigate impairment of ammonia detoxification using the multiscale coupling approach, a PBPK model of ammonia was established (Table S3 in Text S1). To determine rates of ammonia formation and consumption, an equilibrium concentration of 29 µM in venous plasma was considered within the PBPK model, which is the normal level in healthy humans [48]. Three model parameters describing ammonia production and excretion were identified using above physiological information: An overall ammonia production rate of 0.694 µmol/L/min was estimated as well as macroscopic liver and kidney clearance rates of 0.163 µmol/L/min and 0.530 µmol/L/min, respectively. Next, the ammonia PBPK model and the metabolic network were combined by using direct coupling. In particular, the PBPK model was simulated for one time step to calculate the new concentrations and the corresponding liver clearance rate, which could then be used as a new upper bound in the next FBA step. Maximization of ammonia production was used as objective function, which was constrained by substrate availability, exchange rates calculated with the PBPK model as well as enzymatic deficiencies accompanying UCD. At a steady state ammonia concentration of 29.02 µM in the venous blood, the liver cell showed an intracellular ammonia concentration of 25.78 µM (Figure 4 A). In the metabolic network of the hepatocyte, an ammonia uptake flux of 0.163 µmol/L/min was calculated, while the production rates of urea, glutamine and alanine were 0.070 µmol/L/min, 0.008 µmol/L/min and 0.002 µmol/L/min, respectively (Figure 4 B). The demands of glucose and oxygen are in agreement with previous results [26].

With the combined multiscale model, we simulated pathogenesis of urea cycle disorder (UCD) resulting in a reduced ammonia detoxification capacity. Pathogenesis of UCD was exemplarily assumed as a linear decrease of the enzyme activity of ornithine transcarbamylase (OTC), leading to complete impairment of the enzyme (Figure 4 A) [56]. Simultaneously, the glutamine and alanine production rates are increased fourfold above the nominal glutamine and alanine production rates [57]. Hence, while the enzyme activity of OTC is decreased, the maximum activity of glutamine and alanine synthesizing enzymes is increased. Glutamine and alanine production is supposed to increase at 6 h after the onset of UCD, assuming a delay due to transcription and translation initiation.

With the coupled model and the above described constraints, simulation was performed within a time range of 21 days. After 24 h, UCD starts developing which led to a decreasing urea production rate after 30 h. At the same time, the glutamine and alanine fluxes began to rise and the rate of ammonia uptake began to decrease. In the new steady state (after 6.5 days), ammonia uptake rate was 0.072 µmol/L/min (Figure 4 B) and glutamine and alanine production rates were 0.033 µmol/L/min and 0.007 µmol/L/min, respectively (Figure 4 C). The new venous ammonia concentration was 33.99 µM, while the liver concentration was 30.27 µM (Figure 4 A).

**Figure 5:**
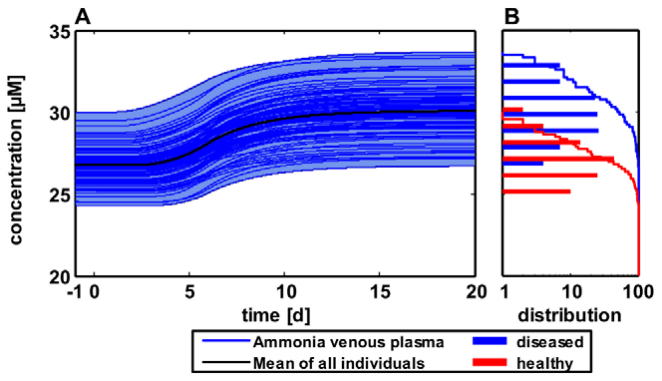
Determining the influence of inter-individual variability during development of a urea cycle disorder. (**A**) Simulated venous plasma concentration profiles of ammonia in 100 individuals during development of a urea cycle disorder (single profiles and mean). (**B**) The distribution of ammonia concentrations as well as the cumulative sums in healthy and diseased individuals are significantly different (p<0.001).

Above model is a representation for an average patient. Since the model, however, structurally includes many potential causes for inter-individual variability at a large level of mechanistic detail, it may be used to analyze pathogenesis on a populations scale as well. Such individual differences may include physiology [58], protein expression [20] or even nutrition [59]. In order to exemplarily describe the effect of inter-individual variability, 100 individuals were simulated based on randomly perturbed production and clearance rates, respectively (assuming 10% standard normal distribution relative to the mean patient, Figure 5 A, Table S4 in Text S1). The distribution of ammonia concentrations in healthy and diseased individuals together with their cumulative sums underlines the inter-individual variability during UCD pathogenesis (Figure 5 B). Performing the Kolmogorov-Smirnov test provided evidence that the distributions in healthy and diseased state differs significantly (p<0.001), making ammonia concentration a quantitative biomarker for OTC deficiency. By performing the population simulation, the results demonstrate that the mere consideration of single individuals may induce misleading diagnoses when specific patient subgroups such as obese, elderly or diseased individuals are to be investigated. Only by population simulations of comprehensive mechanistic models it may become possible to mechanistically discriminate the different, potentially counter-current factors such as high ammonia production rates and low clearance rates.

### Paracetamol toxication

Our final example deals with drug-induced toxication by addressing downstream effects of drug dosing on metabolic functionality at the cellular level. We here chose overdosing of paracetamol, one of the most common reasons for liver failure [60] and poisoning [61]. Paracetamol is generally considered to be an inhibitor of prostaglandin synthesis and is widely used for reducing pain (analgesic) and fever (antipyretic) [62]. At higher doses, paracetamol can cause severe hepatotoxic effects leading to acute liver failure and liver necrosis [63]. Paracetamol is metabolized by three main pathways: (i) glucuronidation, (ii) sulfation and (iii) N-hydroxylation by cytochrome P450 2E1 (CYP2E1) [64]. The corresponding metabolites of the three pathways are (i) paracetamol glucuronide (PG), (ii) paracetamol sulfate (PS) and (iii) N-acetyl-p-benzoquinone imine (NAPQI). In particular, NAPQI, the hydroxylation product, is relevant for consideration of paracetamol toxication since it is highly reactive and toxic [63]. At therapeutic doses, NAPQI is almost immediately detoxified by glutathione (GSH) conjugation. After reacting with GSH, NAPQI is further degraded in the gut and the kidneys and is excreted as paracetamol cysteine (AC) and mercapturic acids [65]. Paracetamol is metabolized primary into PG and PS. At higher doses, however, the pathways synthesizing PG and PS become saturated causing more NAPQI to be produced [64]. In this case, GSH is depleted almost completely (up to 80%) by the detoxification of NAPQI such that the excess of NAPQI accumulates in the liver and the body. The free NAPQI then binds covalently to proteins forming protein adducts which are considered to be one cause for hepatotoxicity of paracetamol [63]. Taken together, the metabolic impact of paracetamol overdose is described by the reduced activity of three enzymes N-10-tetrahydrofolate dehydrogenase (THFDH, up to 25%), glutamate dehydrogenase (GDH, up to 25%) and mitochondrial ATP-Synthetase (ATPS, up to 60%), respectively, and the depletion of GSH by up to 80% [66].

To quantify the metabolic impact of perturbations in the enzyme activities on whole liver functionality, indirect coupling of a paracetamol PBPK model and HepatoNet1 was used to determine the effect of a paracetamol overdose on a large amount of functional liver objectives. HepatoNet1 was previously validated with 123 physiological functions which represent essential tasks for liver metabolism [26]. In particular, 67 of the 123 presented objectives have been tested with three specific sets of extracellular metabolites, which were therefore used as a core set for network validation [26]. To quantify the impact of paracetamol-induced liver failure, we tested the extent by which the value of each of the 67 objective functions is decreased during a paracetamol overdose, thus quantifying hepatic network robustness towards external perturbations [67].

**Figure 6:**
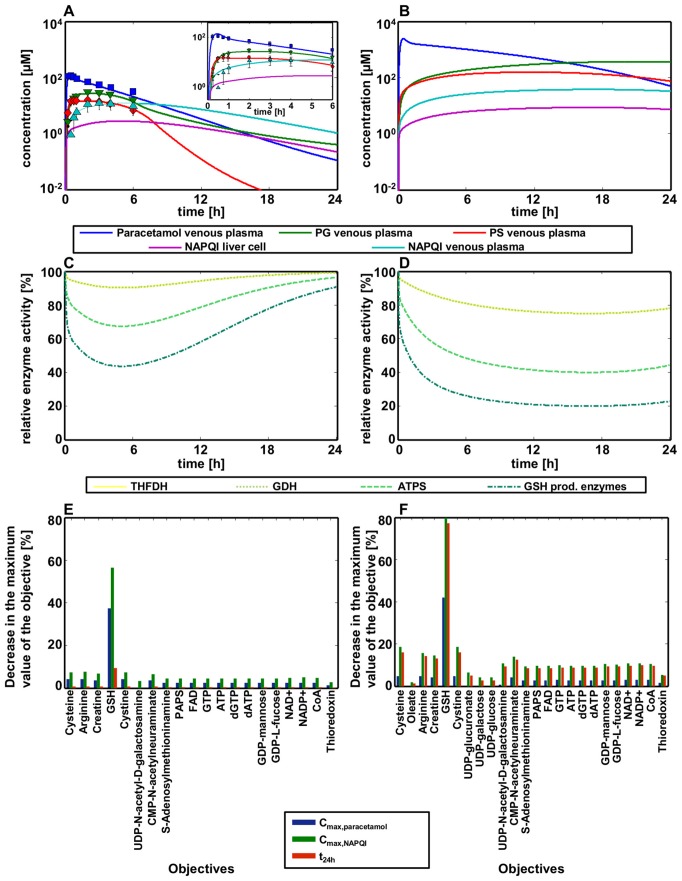
Effect of a therapeutic dose and a toxic overdose of paracetamol on liver functionality. (**A**) Simulated venous plasma concentration profiles of paracetamol and its three metabolites after an paracetamol dose of 1 g are in agreement with experimental PK data [65]. (**B**) Prediction of venous plasma concentration profiles of paracetamol and its three metabolites after a paracetamol dose of 15 g based on the 1 g application model. (**C, D**) Relative enzyme activity of the three impaired enzymes THFDH, GDH, ATPS and the GSH depletion are also implemented as enzyme inhibition after the application of 1 g (C) and 15 g (D) paracetamol, respectively. (**E, F**) Effect of the paracetamol doses of 1 g (E) and 15 g (F), respectively, on liver functionality at three different time points. Bars represent the decrease of maximum values of every objective function which undergoes a change in its maximum value.

We started our analyses with the development of PBPK models of the parent drug paracetamol the three metabolites (Table S5 in Text S1). Oral administration of 1 g paracetamol was considered first. Eight model parameters were identified for the paracetamol model, three for each the PG and the PS model and four for the NAPQI model by comparison of computational simulations with the corresponding experimentally measured venous blood concentrations for all four compounds [65] (Section parameter identification in Text S1, Table S5 in Text S1). Notably, the parameters either characterize physicochemistry of the compounds or describe the physiology of the individuals such that prior knowledge is implicitly included (see Materials and Methods). Moreover the four models are highly interlinked by the underlying mass-balances thereby reducing the systemic degree of freedom significantly. Since the metabolization of NAPQI into AC cannot be quantified due to missing literature information, it was assumed that the AC concentration is equivalent to the NAPQI concentration. After parameter adjustment, the PBPK simulations of paracetamol, PG, PS and NAPQI described the experimental data with excellent agreement (Figure 6 A, Table S6 in Text S1). Subsequently, the PK of the four compounds after a lethal dose of 15 g paracetamol was predicted (Figure 6 B, Table S7 in Text S1).

In order to investigate changes in metabolic functionality following a paracetamol overdose, FBA was first performed to determine the optimal value of every objective function and to quantify all fluxes in the healthy, untreated reference individual. Subsequently, the inhibition of enzymes by paracetamol and its active metabolites was implemented as additional constraints on the flux values. For every inhibited enzyme the flux value was fixed as the product of the value in the healthy state and the remaining relative enzyme activity (Equation 6). The *K_i_* values were calculated by assuming that the maximum concentration of NAPQI after a lethal dose of 15 g paracetamol induces the maximum enzyme inhibition as described above. In order to calculate *K_i_* values for paracetamol and all three metabolites, substrate concentrations were assumed to be equal to *K_m_* (Equations S7–S12 in Text S1). Therefore, the time-resolved relative enzyme activities (Figure 6 C, D) could be calculated by:
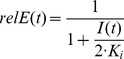
(6)FBA was then performed for every objective function with these additional constraints. Since NAPQI is not naturally produced in the liver, the molecular interaction of exogenous NAPQI with endogenous GSH cannot be implemented in HepatoNet1 without structural network modifications. Hence, the production of NAPQI as an exogenous compound is simulated in the PBPK model as described above. The depletion of GSH was considered rather phenomenological by indirect coupling, thereby inducing a decrease in enzyme activity which is linked by indirect coupling to the intracellular NAPQI concentration. Neither metabolite pool size of GSH nor regulatory effects following depletion of GSH can be mechanistically considered in stoichiometric models such as HepatoNet1. Therefore, all reactions producing GSH (Table S8 in Text S1) in HepatoNet1 were identified and inhibited as described above, such that GSH consuming reactions are limited. The change in liver functionality was calculated as the difference between the maximum values of the objective functions in the healthy state and in the case of a paracetamol overdose. Three distinct time points were considered which included time of peak concentrations of paracetamol (t_max,paracetamol_) and NAPQI (t_max,NAPQI_), respectively as well as time of trough concentrations at 24 h after drug administration (t_24h_) (Figure 6 E, F). Out of the 67 objective functions considered, 20 objective functions underwent a change after the application of 1 g paracetamol with respect to the untreated state, while 24 objective functions underwent a change after the application of 15 g paracetamol. Almost all optimal values were at reference values after 24 h for a 1 g dose of paracetamol, while all affected objectives remained severely decreased after 24 h for a 15 g dose.

**Figure 7:**
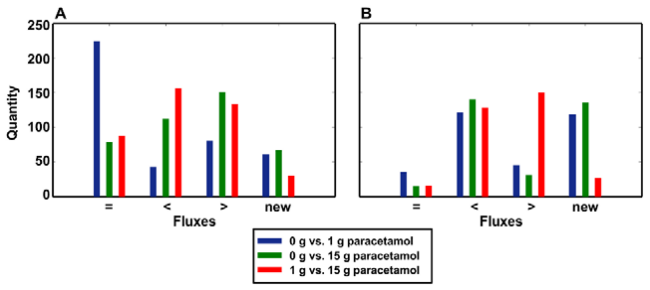
Comparison of two specific liver functions (production of oleate and cysteine, respectively). Only fluxes which are nonzero at least in one of the three cases are compared. The number of fluxes which remain at their original values ( = ), become smaller or higher (<, >) or are non-zero (new) after application of 1 g and 15 g of paracetamol are indicated.. (**A**) Changes in fluxes after application of 1 g and 15 g paracetamol for production of oleate. (**B**) Changes in fluxes after application of 1 g and 15 g paracetamol for production of cysteine.

We next analyzed, whether the flux underlying the different optimal values changed during the application of different doses of paracetamol. This could explain why more objective functions are affected after the toxic dose of paracetamol, which is to be expected by network robustness [67]. As an example of an objective function which is only affected after the toxic dose, the production of oleate showed a greater robustness to the metabolic effect of paracetamol, as many active fluxes underlying this objective function remained unchanged after the application of 1 g paracetamol (54.8%) (Figure 7 A). Likewise, the maximum value of the objective was only slightly decreased after the application of 15 g paracetamol. In contrast, paracetamol administration showed a greater effect to the maximum value of cysteine production, as the maximum value was already decreased after an application of 1 g paracetamol compared to the untreated reference state. Furthermore, only 11.1% of the fluxes underlying this objective function remained unchanged after paracetamol administration and many new fluxes became active, suggesting that flux rerouting [68] was used to compensate the inhibition in the impaired metabolic pathway (Figure 7 B).

## Discussion

We here integrate genome-scale metabolic networks, comprising thousands of biochemical reactions, into PBPK models to analyze metabolism at the level of the human organism. The approach is based on dFBA [31] which is used to simulate stationary stoichiometric metabolic networks at the cellular level in combination with ODE-based PBPK models. This semi-continuous approach uses the metabolic exchange rates as input at the organism level and vice versa the drug concentrations at the whole-body scale to calculate upper bounds for the enzyme activities in the metabolic network.

Our approach combines well-established computational modeling approaches from different biological scales. At the organism scale, standardized PBPK models which are routinely used in pharmaceutical drug development provide a generalized description of the distribution of substances within the human body. At the cellular scale, metabolic network reconstructions represent core building blocks of bottom-up systems biology which describe fundamental cellular biochemistry. Our coupling approach therefore provides a generic framework for a wide range of possible applications. Notably, all these applications can be addressed without the need for further model curation or modification.

To illustrate our approach, we exemplarily integrate HepatoNet1 [26], a genome-scale metabolic model of a human hepatocyte into the liver tissue of a standardized PBPK model [11], [69]. As in reality, metabolization processes at the organism level therefore result from the biochemical reaction within the hepatocyte. To illustrate the broad applicability of our approach, three case studies are presented addressing prototypical medical and pharmaceutical questions.

In a first application example we investigate a multiscale PK/PD approach, where indirect coupling is used to mechanistically describe the pharmacological effect of the purine analogue allopurinol on the biosynthesis of uric acid. The PK of the exogenous drug as well as its resulting downstream PD effect on the formation of endogenous metabolites are quantified allowing a comprehensive evaluation of drug safety and drug efficiency. For multiple oral administrations of 200 mg allopurinol we predict a 69.3% decrease of uric acid concentration in the venous plasma which is in quantitative agreement with clinical data [48]. Since the corresponding PK/PD model has been established with data from single dosings of allopurinol [43], this accurate prediction of the long term therapeutic effect convincingly illustrates the predictive power of our approach.

The identification of quantitative biomarkers for metabolic disorders is the second application example. As a complement to classical qualitative biomarker identification [70], our approach provides quantitative information in terms of specific concentration profiles by simulating the distribution of the affected compounds at the organism level. This enables a mechanistic description of metabolic disorders such as IEMs in blood plasma and further biofluids. As an exemplary case study for quantitative biomarker identification, we investigate impaired ammonia detoxification resulting in an increase of 17% of ammonia in blood plasma. We next simulate virtual populations of healthy individuals and patients by varying anatomical and physiological parameters according to prior statistical information [58]. Despite a considerable level of inter-individual variability in both subgroups, we demonstrate that the difference in ammonia plasma levels between both subgroups is statistically significant. Notably, the structural complexity of PBPK models together with the prior physiological and anatomical information included helps to explain counter-intuitive behavior during disease progression in individual patients, since many relevant co-factors are mechanistically presented in the model structure itself. This is important in clinical practice, since in addition to diagnostics of key metabolites other contributing factors are structurally considered in the model such that variability of the healthy reference state can be mechanistically quantified.

Drug-induced toxication following paracetamol overdose is the third application example. We demonstrate how paracetamol impairs metabolic capacity by affecting a broad range of different metabolic functions. The specific metabolic impact is illustrated for a therapeutic (1 g) and a toxic (15 g) dose of paracetamol. The results show a larger metabolic impact on single hepatic functions following the toxic dose of paracetamol. Also, more metabolic functions are affected after the higher dose (24 vs. 20 functions). Since a considerable number of fluxes is affected, we conclude that paracetamol toxication induces flux rerouting which is used by the hepatocyte to compensate for network perturbations thereby conferring cellular robustness. Notably, this effect which has been shown before for microorganisms [68] is distributed over the whole metabolic network and can only be investigated with genome-scale models.

Describing the impact of a compound on the body and the cell is the fundamental question in pharmacodynamics. Classical approaches describe this interference with rather phenomenological models [14]. By replacing the cellular space with metabolic network models at genome-scale, our approach describes cellular processes at a much higher level of detail. To quantify specific metabolic states we use objective functions which have been used before to verify functional capacity of the liver [26]. It should be noted that such functions do not allow to identify actual intracellular flux distributions unlike shown before for microorganisms [71]. In contrast, the flux space is evaluated qualitatively in the face of external or internal perturbations. Additionally constraining FBA optimization with kinetic rates simulated with the PBPK models, however, provides an important transfer of physiological information in-between both model scales. If it would be possible to quantify intracellular flux distributions in mammalian cells as well, further algorithms [72] could be used to further asses and characterize the overall flux space. This may also involve the consideration of additional experimental information such as omics data [73] or inclusion of regulatory information [74].

Taken together, the presented approach integrating organ-specific metabolic networks into PBPK models provides many opportunities for scientific research, clinical applications and drug development as outlined by the prototypical examples discussed above. It is only by such multiscale models that a mechanistic understanding of organ dysfunction and disease etiology at a system level will be achieved. This will be greatly supported by the reconstruction of further genome-scale metabolic networks which will become available in the future [7]. Structurally, the metabolic networks provide a template onto which the genetic predisposition of a patient can be mapped. Together with specific physiological information this may someday allow model-based optimizations of risk-benefit profiles in personalized medicine.

## Materials and Methods

### PBPK modeling

At the whole-body level, PBPK modeling quantitatively describes all ADME-related processes of endogenous and exogenous compounds within mammalian organisms [10], [11], [12], [13]. In contrast to the rather descriptive consideration of PK in classical compartmental models, PBPK models include a detailed representation of physiological processes within an organism which is based on prior knowledge and information. The underlying model structure, which connects the various tissues compartments and the vascular system, is based on generic distribution models and quantifies the mass transfer across the different sub-compartments. Parameters of distribution models are automatically derived from the physicochemistry of the compounds such as the molecular weight or the lipophilicity [15], [16], [17], [18], [19]. Parameters describing the physiology of an organism such as organ volumes, blood flow rates or tissue composition are obtained from collections of physiological data and are available in the internal PBPK software database. Due to this large amount of prior structural and physiological information, the number of independent parameters which need to be identified during model development is small (usually less than 10). Notably, there is a clear separation between physiological parameters which refer to the organism in which a compound is distributed and compound specific parameters which specifically describe the properties of the substance itself. Thereby, compound parameters only need to beadjusted in a narrow range since literature information already is available. In contrast, physiological parameters have to be identified individually.

### Creating PBPK models with PK-Sim and MoBi

The PBPK models considered in this study were all built with the software tools PK-Sim and MoBi for which academic licenses are available free of charge (Section software information in Text S1) and which have been explained in detail before [11], [69]. PBPK models were created for parent substances as well as for all metabolites. Compound specific parameters were used in each case to parameterize the underlying structure of the PBPK model. In all models, we considered mean individuals. The anthropometric information regarding age, weight and height further specifies the selection of physiological parameters as provided in the software. This allows a specific parameterization of the PBPK model, since the model contains only few independent parameters as described above.

Case-specific metabolization and clearance processes represented by Michaelis-Menten kinetics or first order reactions and according to literature information are additionally defined. For the corresponding kinetic parameters, parameter adjustment is important since (1) usually less literature data is available and (2) these parameters often have significant sensitivity although the overall dynamic behavior of the PBPK models is robust (Figure S3 in Text S1).

For the examples of allopurinol treatment and paracetamol toxication, PBPK models in PK-Sim are exported to MoBi where they are further modified. PBPK models of the parent drug and its metabolites are connected such that the distribution of all compounds is described simultaneously. In this case, the rate of the clearance reaction in the parent drug model is set as the input into the metabolite model.

### Stoichiometric network models

Stoichiometric models provide a mathematically formal way to capture the basic biochemistry of cellular metabolism into an analytical framework. Assuming steady state of the system, all intracellular metabolites can be balanced in linear systems of equations, which are usually underdetermined since they encompass much more unknown reaction rates than linear independent mass balance equations. Flux distributions can be identified with stoichiometric models by using FBA [35], [75], [76]:
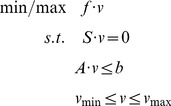
(4)Here, *f · v* (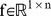, 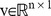) corresponds to the objective function which reflects a rationale of cellular function, *S* (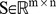) denotes the stoichiometric matrix of the metabolic network (including *m* metabolites and *n* reactions) and *v* represents the intracellular flux distribution. The overall solution space is confined by a set of additional constraints (*A·v≤b*) which represent for example substrate availability.

### HepatoNet1

To illustrate our approach, we here exemplarily use HepatoNet1, a tissue-specific network reconstruction of hepatic metabolism [26]. HepatoNet1 consists of 777 metabolites and 2539 reactions and is divided into six intra- and two extracellular compartments. Its general network structure was validated by verifying the accomplishment of 123 biochemical objectives representing metabolically feasible functional modes. Hence, the network was constructed specifically to examine distinct metabolic processes of the liver and provides a structural platform for mechanistic studies of tissue-specific physiological functions [26].

## Supporting Information

Text S1 Important additional texts, tables, equations and figures.
(PDF)
