# Supplementary material for: Integrating Cellular Metabolism into a Multiscale Whole-Body Model
Source: PLoS Comput Biol. 2012 Oct 25;8(10):e1002750. doi: 10.1371/journal.pcbi.1002750 (PMC3486908; doi:10.1371/journal.pcbi.1002750)
Supplement: Text S1 — Important additional texts, tables, equations and figures. (PDF) [file pcbi.1002750.s001.pdf]

Integrating cellular metabolism into a multiscale whole-body model

Supporting information: Text S1

Software information

The PBPK models were built using the commercial software tool PK-Sim® Version 4.2 (Bayer Technology Services GmbH, Leverkusen, Germany) [1,2,3,4]. PK-Sim® generated PBPK models were exported and modified in MoBi® (version 2.2; Bayer Technology Services) as described further below. All optimizations and batch mode simulations were carried out using Matlab (version 7; MathWorks, Natick, MA) and the MoBi® Toolbox for Matlab (version 2.0; Bayer Technology Services).

Parameter identification

Optimizations were based on an fminseach algorithm as provided by the MoBi® Toolbox for Matlab. In order to identify solutions close to global optima [5], first, repetitive optimizations were performed for each model version to find satisfying sets of start values, based on randomly distributed starting guesses (Monte Carlo). Subsequently, fminsearch was performed to find optimal solutions. The root mean square deviation (RMSD) relative to the various experimental data was considered as objective function for all optimizations.

Tables

Allopurinol treatment

Table S1

Parameters used for initializing the PBPK model of allopurinol and oxypurinol. Model parameters represent the adjusted values.

Parameter	Compound	Organ	Literature parameter	Reference	Model parameter	Unit
molecular weight	allopurinol		136.11	CID: 2094[1]		g/mol
lipophilicity (LogP)			-0.5	CID: 2094[1]	-0.51	
unbound fraction *(*fu*)*			99	[6,7]	98	%
CL_{spec}		liver			0.0088	L/min kg
P_{int}					0.0003	cm/min
molecular weight	oxypurinol		152.11	HMDB[2]		g/mol
LogP			-0.9	HMDB[2]	-0.07	
fu			99	[6]	90.11	%
CL_{spec}		liver			0.0003	L/min kg
CL_{spec}		kidney			0.0001	L/min kg

[1] http://pubchem.ncbi.nlm.nih.gov

[2] http://www.hmdb.ca/metabolites/HMDB00786

Table S2

Parameters used for initializing the PBPK model of uric acid. Model parameters represent adjusted values.

Parameter	Compound	Organ	Literature parameter	Reference	Model parameter	Unit
molecular weight	uric Acid		168.11	HMDB[3]		g/mol
LogP			-2.17	HMDB[3]		
fu			99	no reference available[4]		%
pKa 1			5.4	[8]		
pKa 2			10.3	[8]		
$CL_{spec,healthy}$		kidney			1.18e-4	L/min kg
$CL_{spec,gouty}$		kidney			7.46e-5	L/min kg
production rate		liver			2.24	µmol/min

[3] http://www.hmdb.ca/metabolites/HMDB00289

[4] assumption as start value for fitting!

Ammonia detoxification

Table S3

Parameters used for initializing the PBPK model of ammonia.

Parameter	Compound	Literature parameter	Unit	Reference
molecular weight	ammonia	17.03	g/mol	HMDB[5]
LogP		0.23		HMDB[5]
fu		99.00	%	no reference available[6]

Table S4

Calculated standard normal distributions of liver clearance and production rate.

Parameter	Mean	Range	Distribution N(μ, σ)
clearance liver	0.1632	[0.1469 0.1795]	N(0.1632, 0.0059)
production rate	0.6939	[0.6245 0.7633]	N(0.6393, 0.0252)

[5] http://www.hmdb.ca/metabolites/HMDB00051

[6] assumption as start value for fitting!

Paracetamol toxication

Table S5

Parameters used for initializing the PBPK model of paracetamol and its metabolites. Model parameters represent adjusted values.

Parameter	Compound	Organ	Literature parameter	Reference	Model parameter	Unit
molecular weight	paracetamol		151.16	HMDB[7]		g/mol
LogP			0.46	HMDB[7]		
fu			80.00	[9]		%
pKa			9.50	[10]		
CL_{spec}		kidney			0.046	L/min kg
P_{int}					0.001	cm/min
$v_{max\text{-}Gluc}$		liver			13.888	µmol/min kg tissue
$K_{m\text{-}Gluc}$		liver			131.150	µmol/L
$v_{max\text{-}Sulf.}$		liver			20.199	µmol/min kg tissue
$K_{m\text{-}Sulf.}$		liver			320.680	µmol/L
$v_{max\text{-}NAPQI}$		liver			2.215	µmol/min kg tissue
$K_{m\text{-}NAPQI}$		liver			46.742	µmol/L
molecular weight	PG		327.29	HMDB[8]		g/mol
LogP			-0.68	HMDB[8]		
fu			90.00	[9]		%
CL_{spec}		kidney			9.058	L/min kg
v_{max}		liver			3.133	µmol/min kg tissue
K_m		liver			0.001	µmol/L

[7] http://www.hmdb.ca/metabolites/HMDB01859

[8] http://www.hmdb.ca/metabolites/HMDB10316

molecular weight	PS		231.33	CID: 83939[9]		g/mol
LogP			0.10	CID: 83939[9]		
fu			40.00	[9]		%
CL_{spec}		kidney			0.002	L/min kg
v_{max}		liver			1.876	µmol/min kg tissue
K_m		liver			0.107	µmol/L
molecular weight	NAPQI		149.15	CID: 39763[9]		g/mol
LogP			0.10	CID: 39763[9]		
fu			2.00	no reference available[10]	26.162	%
CL_{spec}		kidney			0.002	L/min kg
v_{max}		liver			7.868	µmol/min kg tissue
K_m		liver			32.513	µmol/L

[9] http://pubchem.ncbi.nlm.nih.gov

[10] assumption as start value for fitting!

Table S6

Pharmacokinetic parameters describing the simulation results after the application of 1 g paracetamol.

Compound	Parameter	Value	Unit
paracetamol	C_{max}	140.42	µM
	t_{max}	28.10	min
	$AUCt_{end}$	23800.00	µmol h/L
PG	C_{max}	26.78	µM
	t_{max}	139.80	min
	$AUCt_{end}$	10400.00	µmol h/L
PS	C_{max}	14.91	µM
	t_{max}	45.10	min
	$AUCt_{end}$	5030.00	µmol h/L
NAPQI - venous blood	C_{max}	12.12	µM
	t_{max}	330.70	min
	$AUCt_{end}$	9300.00	µmol h/L
NAPQI - liver cell	C_{max}	2.78	µM
	t_{max}	304.50	min
	$AUCt_{end}$	2120.00	µmol h/L

Table S7

Pharmacokinetic parameters describing the simulation results after the application of 15 g paracetamol.

Compound	Parameter	Value	Unit
paracetamol	C_{max}	2435.70	µM
	t_{max}	29.50	min
	$AUCt_{end}$	932000.00	µmol h/L
PG	C_{max}	368.78	µM
	t_{max}	1127.00	min
	$AUCt_{end}$	378000.00	µmol h/L
PS	C_{max}	156.49	µM
	t_{max}	698.80	min
	$AUCt_{end}$	174000.00	µmol h/L
NAPQI - venous blood	C_{max}	37.82	µM
	t_{max}	1035.90	min
	$AUCt_{end}$	43100.00	µmol h/L
NAPQI - liver cell	C_{max}	8.62	µM
	t_{max}	1013.60	min
	$AUCt_{end}$	9990.00	µmol h/L

Table S8

Numbers of blocked reactions of GSH production for the simulation of GSH depletion together with their corresponding EC numbers and enzyme designations.

Reaction Nr [11]:	EC/ TCDB Number [11]	Enzyme[11]
r21	EC:1.8.1.7	glutathione-disulfide reductase
r22		
r25		
r26		
r87	EC:1.11.1.9	glutathione peroxidase
r88		
r89		
r129	EC:3.4.11.4	tripeptide aminopeptidase
r130		
r131	EC:6.3.2.3	Glutathione synthase
r885	TCDB:2.A.29.2.7	Mitochondrial dicarboxylate carrier
r1377	EC:4.4.1.22	S-(hydroxymethyl)glutathione synthase
r1379	EC:3.1.2.12	S-formylglutathione hydrolase

[11] http://www.brenda-enzymes.info/

Equations

In the following, few important transformations of Michaelis-Menten equations for the calculation of the relative enzyme activity (*relE*) are shown.

Allopurinol treatment

For unknown K_i, but known IC50 and two inhibitors:

$$v(t) = \frac{v_{\max} \cdot S}{S + K_m \cdot (1 + \frac{I_1(t)}{K_{i1}} + \frac{I_2(t)}{K_{i2}})} \quad \text{(S1)}$$

$$v_0 = \frac{v_{\max} \cdot S}{S + K_m} \quad \text{(S2)}$$

$$relE(t) = \frac{v(t)}{v_0} \quad \text{(S3)}$$

S1 & S2 in S3

$$relE(t) = \frac{S + K_m}{S + K_m \cdot (1 + \frac{I_1(t)}{K_{i1}} + \frac{I_2(t)}{K_{i2}})} \quad \text{(S4)}$$

$$K_i = \frac{IC50}{1 + \frac{S}{K_m}} \quad \text{(S5)}$$

S5 in S4

$$relE(t) = \frac{1}{1 + \frac{I_1(t)}{IC50_1} + \frac{I_2(t)}{IC50_2}} \quad \text{(S6)}$$

Paracetamol toxication

For unknown K_i and S, but known relE at I_{max}

$$v(t) = \frac{v_{\max} \cdot S}{S + K_m \cdot (1 + \frac{I(t)}{K_i})} \tag{S7}$$

$$S = K_m \tag{S8}$$

S8 in S7

$$v(t) = \frac{v_{\max}}{2 + \frac{I(t)}{K_i}} \tag{S9}$$

S8 in S2

$$v_0 = \frac{v_{\max}}{2} \tag{S10}$$

S9 & S10 in S3

$$relE(t) = \frac{1}{1 + \frac{I(t)}{2 \cdot K_i}} \tag{S11}$$

Calculation of K_i from S11

$$K_i = \frac{I_{\max}}{2 \cdot (relE_{\max}{}^{-1} - 1)} \tag{S12}$$

Figures

Figure S1

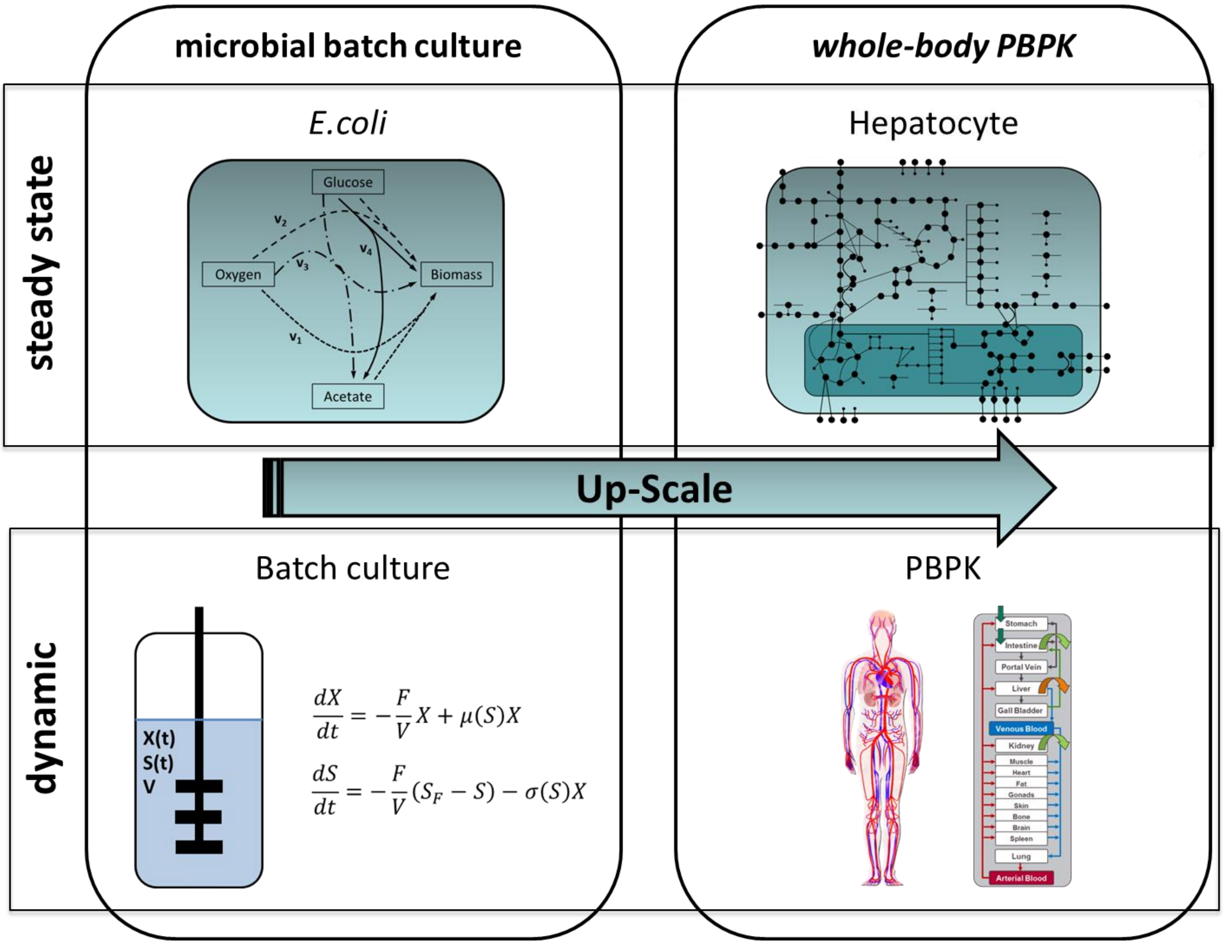

Figure S1. Up-scaling the two layers of the dynamic FBA approach. Instead of a stoichiometric model of central carbon metabolism, a genome scale network of a human hepatocyte, *HepatoNet1* [11], was used in our approach. While in the original approach a batch reactor system of the original approach was considered [12], we applied a whole-body PBPK model to represent the dynamic model of the approach.

Figure S2

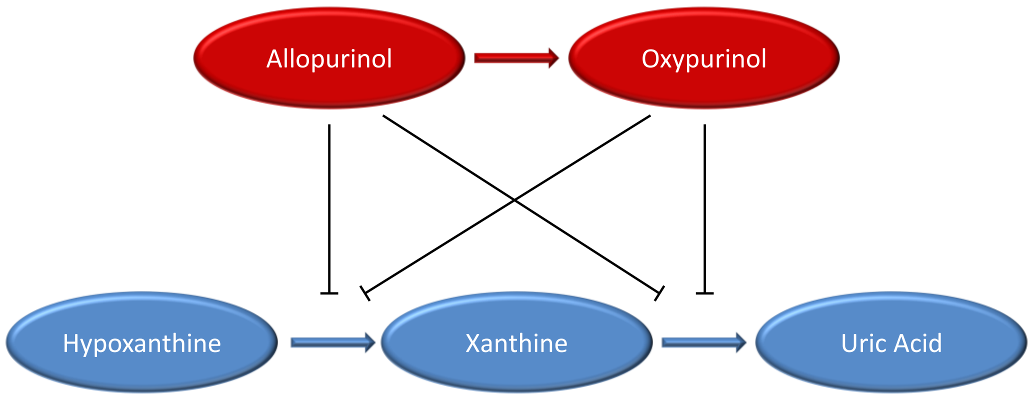

Figure S2. A schematic representation of allopurinol metabolism. All reactions (blue and red arrows) are triggered by the enzyme xanthine-oxidase. Black lines represent mechanism of inhibition of allopurinol and oxypurinol.

Figure S3

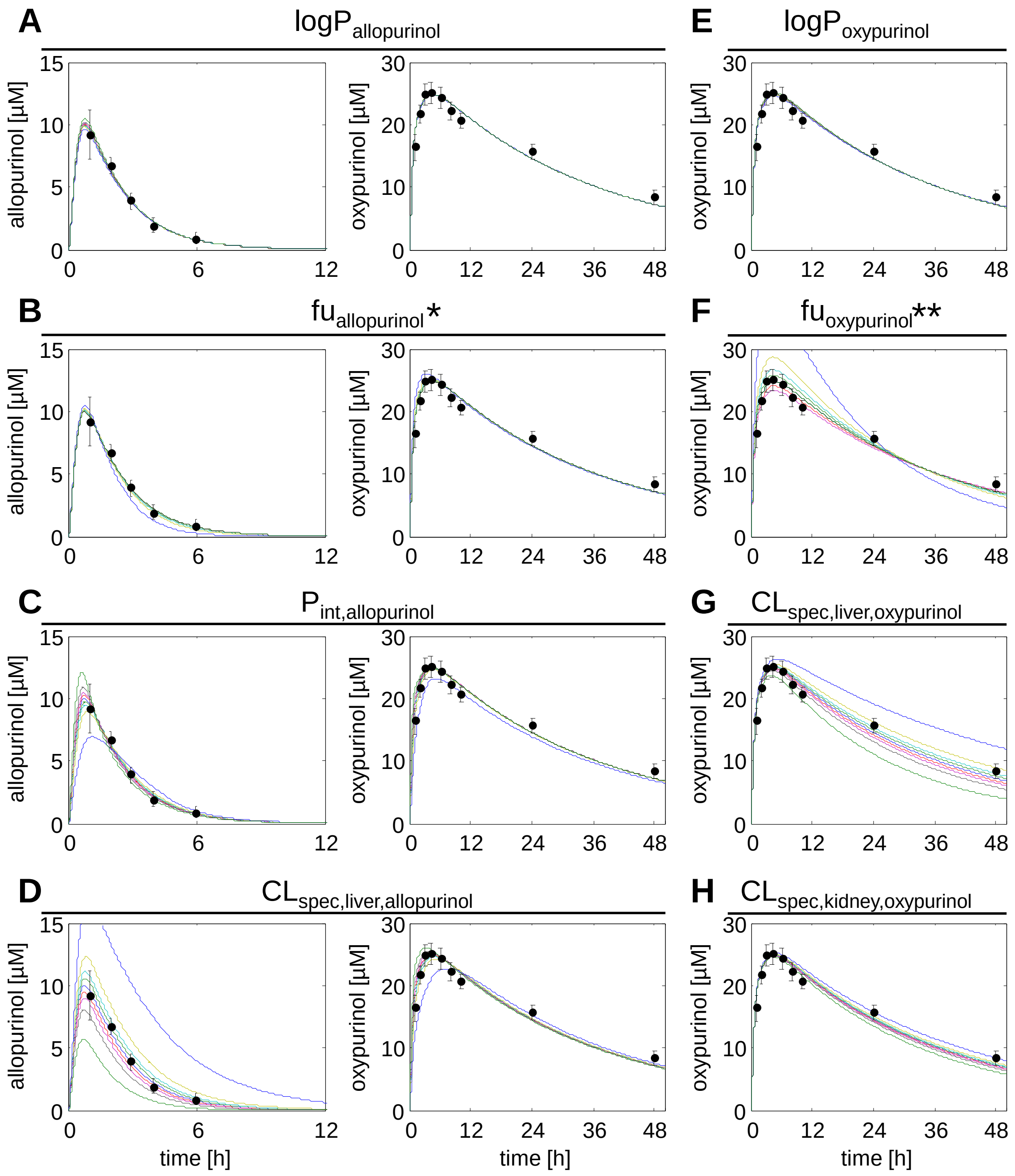

Figure S3. Sensitivity analysis for adjusted parameters in the PBPK model for allopurinol and oxypurinol. Eight parameters have been adjusted to fit the coupled model of allopurinol and its metabolite oxypurinol to experimental data from [6]. logP: lipophilicity, fu: fraction of the drug which is not bound to plasma proteins such as serum albumin, P_{int}: intestinal permeability, which determines the absorbtion of the drug, CL_{spec}: specific clearance constant. Sensitivity analysis has been performed by

modifying the best fit value of each parameter (±5 %, ±10 %, ±20 %, ±50 %). The simulation result is shown for each set of changed parameter (lines) together with experimental data (circles). (**A-D**) Titles represent the parameters which have been changed in the PBPK model for allopurinol. Left figures show PK curves for allopurinol, right figures show PK curves for oxypurinol. (**E-H**) Titles represent the parameters which have been changed in the PBPK model for oxypurinol. Figures show PK curves for oxypurinol.

***** Since the fraction unbound (fu) value is near its upper physiological boundary (100 %) sensitivity analysis could only be performed for –5,-10,-20,-50 %.
****** Since the fraction unbound (fu) value is near its upper physiological boundary (100 %) sensitivity analysis could not be performed for +50 %.

References

1. Eissing T, Kuepfer, L, Becker, C, Block, M, Coboeken, K, Gaub, T, Goerlitz, L, Jaeger, J, Loosen, R, Ludewig, B, Meyer, M, Niederalt, C, Sevestre, M, Siegmund, H-U, Solodenko, J, Thelen, K, Telle, U, Weiss, W, Wendl, T, Willmann, S, Lippert, J (2011) A computational systems biology software platform for multiscale modeling and simulation: integrating whole-body physiology, disease biology, and molecular reaction networks. Front Physio 2.
2. Willmann S, Lippert J, Schmitt W (2005) From physicochemistry to absorption and distribution: predictive mechanistic modelling and computational tools. Expert Opin Drug Metab Toxicol 1: 159-168.
3. Willmann S, Lippert J, Sevestre M, Solodenko J, Fois F, et al. (2003) PK-Sim©: a physiologically based pharmacokinetic 'whole-body' model. Biosilico 1: 121-124.
4. Willmann S, Schmitt W, Keldenich J, Lippert J, Dressman JB (2004) A physiological model for the estimation of the fraction dose absorbed in humans. J Med Chem 47: 4022-4031.
5. Moles CG, Mendes P, Banga JR (2003) Parameter estimation in biochemical pathways: a comparison of global optimization methods. Genome Res 13: 2467-2474.
6. Turnheim K, Krivanek P, Oberbauer R (1999) Pharmacokinetics and pharmacodynamics of allopurinol in elderly and young subjects. Br J Clin Pharmacol 48: 501-509.
7. Murrell GA, Rapeport WG (1986) Clinical pharmacokinetics of allopurinol. Clin Pharmacokinet 11: 343-353.
8. McCrudden FH (2010) Uric Acid: The Chemistry, Physiology and Pathology of Uric Acid and the Physiologically Important Purin Bodies, with a Discussion of the Metabolism In: BiblioBazaar.
9. Morris ME, Levy G (1984) Renal clearance and serum protein binding of acetaminophen and its major conjugates in humans. J Pharm Sci 73: 1038-1041.
10. Prescott LF (1980) Kinetics and metabolism of paracetamol and phenacetin. Br J Clin Pharmacol 10: 291S-298S.
11. Gille C, Bölling C, Hoppe A, Bulik S, Hoffmann S, et al. (2010) HepatoNet1: a comprehensive metabolic reconstruction of the human hepatocyte for the analysis of liver physiology. Mol Syst Biol 6.
12. Mahadevan R, Edwards JS, Doyle FJ, 3rd (2002) Dynamic flux balance analysis of diauxic growth in Escherichia coli. Biophys J 83: 1331-1340.
